# Water Management Fault Diagnosis by Operando Distribution of Relaxation Times Analysis for Anion Exchange Membrane Fuel Cells

**DOI:** 10.1002/advs.202505304

**Published:** 2025-05-14

**Authors:** Haodong Huang, Zijie Zhang, Cailin Xiao, Jiapeng Liu, Zheng Li, Yuting Jiang, Lei Wei, Tianshou Zhao, Francesco Ciucci, Lin Zeng

**Affiliations:** ^1^ Shenzhen Key Laboratory of Advanced Energy Storage Department of Mechanical and Energy Engineering Southern University of Science and Technology Shenzhen 518055 China; ^2^ SUSTech Energy Institute for Carbon Neutrality Southern University of Science and Technology Shenzhen 518055 China; ^3^ School of Advanced Energy Sun Yat‐Sen University Shenzhen 518107 China; ^4^ Chair of Electrode Design for Electrochemical Energy Systems University of Bayreuth Weiherstraße 26 95448 Bayreuth Germany; ^5^ Bavarian Center for Battery Technology (BayBatt) University of Bayreuth Universitätsstraße 30 95447 Bayreuth Germany

**Keywords:** anion exchange membrane fuel cells, distribution of relaxation times, electrochemical impedance spectroscopy, transport resistance, water management faults

## Abstract

Timely and effective fault diagnosis is essential to ensure the reliability and longevity of anion exchange membrane fuel cells (AEMFCs). This study employs operando electrochemical impedance spectroscopy (EIS) measurements and distribution of relaxation times (DRT) analysis to detect water management faults in both anode and cathode electrodes. EIS measurements are performed under diverse operating conditions, revealing three distinct frequency ranges associated with ion transport, charge transfer, and mass transport processes, and elucidating their contributions to voltage loss. Building on these findings, DRT analysis is further applied to explore the behavior and variation of polarization impedance under different water management fault conditions. Compared with the reference case, anode flooding reduces ion transport resistance by up to 37.1%, while increasing charge transfer and mass transport resistances by 61.8% and 219.2%, respectively. Conversely, cathode flooding results in a 33.5% increase in charge transfer resistance, with minimal impact on mass transport resistance. These quantitative insights provide a novel and effective diagnostic tool for distinguishing water management fault types (flooding or drying) and their location (anode or cathode), offering valuable data to support the implementation of water management control strategies that enhance performance and extend the lifespan of commercial AEMFC stacks.

## Graphical abstract

An in situ characterization platform is established for water management and fault diagnosis in AEMFCs by employing the relaxation time method to analyze electrochemical impedance spectra. This approach identifies the characteristic frequency ranges of ionic, charge, and reactant transport resistances, enabling quantitative diagnosis of water transport failures and elucidation of the associated degradation mechanisms.

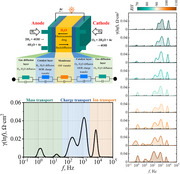

## 1 Introduction

Due to their high efficiency and low emissions, hydrogen‐fed fuel cells have emerged as one of the most promising energy conversion devices for addressing the urgent challenges of global warming and environmental pollution.^[^ ^1^, ^2^, ^3^ ^]^ Among these technologies, anion exchange membrane fuel cells (AEMFCs) have gained considerable attention due to their merits, such as rapid oxygen reduction reaction (ORR) kinetics and high energy conversion efficiency.^[^ ^4^, ^5^, ^6^ ^]^ However, despite these advantages, the limited reliability and durability of AEMFCs remain substantial barriers to their widespread commercialization. One of the major challenges is the water management faults during fuel cell operation, including flooding and drying, which block mass transport and accelerate membrane degradation. These issues critically impair performance and significantly shorten the fuel cell's lifespan.^[^ ^7^, ^8^, ^9^ ^]^ Therefore, in addition to material advancements, developing effective fault diagnosis methods is crucial to enhancing durability and minimizing maintenance costs.

Various diagnostic techniques are employed to ensure the reliable operation of fuel cells, broadly classified into electrochemical and physicochemical methods. Electrochemical techniques include polarization curves,^[^ ^10^ ^]^ electrochemical impedance spectroscopy (EIS),^[^ ^11^, ^12^ ^]^ and cyclic voltammetry.^[^ ^13^ ^]^ On the other hand, physicochemical methods encompass pressure measurement,^[^ ^14^ ^]^ gas chromatography,^[^ ^15^ ^]^ neutron imaging,^[^ ^16^ ^]^ magnetic resonance imaging,^[^ ^17^ ^]^ and temperature mapping.^[^ ^18^ ^]^ Among these diagnostic tools, EIS stands out as a well‐established and widely utilized approach in fuel cell research. Applying an AC perturbation at varying frequencies allows EIS to distinguish polarization processes with different time constants in the impedance spectrum. This technique provides critical insights into major sources of performance losses within the fuel cell, such as charge transfer resistance, mass transport resistance, and ohmic resistance.^[^ ^19^, ^20^ ^]^

Previous studies have demonstrated a strong correlation between resistance measured by EIS and cell voltage over prolonged operation, underscoring the utility of EIS in diagnosing faults and predicting performance degradation.^[^ ^21^, ^22^ ^]^ A significant challenge in utilizing EIS lies in interpreting impedance spectra to accurately quantify the various sources of performance losses within the fuel cell, thus facilitating the identification and diagnosis of operational faults. Traditionally, this has been addressed through equivalent circuit models (ECM), which use circuit elements such as resistors, capacitors, and Warburg elements to represent processes like charge transfer, double‐layer capacitance, and diffusion. For instance, S. Tant et al.^[^ ^23^ ^]^ and Heesoo Choi et al.^[^ ^24^ ^]^ employed EIS to diagnose proton exchange membrane fuel cell (PEMFC) flooding, observing increased impedance under flooding conditions compared to optimal operating conditions. However, the ECM approach requires prior system knowledge, and fuel cell performance is influenced by multiple factors, often necessitating complex circuit configurations for accurate data fitting.^[^ ^25^, ^26^ ^]^ This complexity can lead to multiple circuit configurations producing similar impedance responses, complicating physical interpretation.^[^ ^27^, ^28^ ^]^ To overcome these limitations, this study pioneers the use of model‐free DRT analysis for interpreting EIS data to diagnose water management faults in AEMFCs.

**Figure 1:**
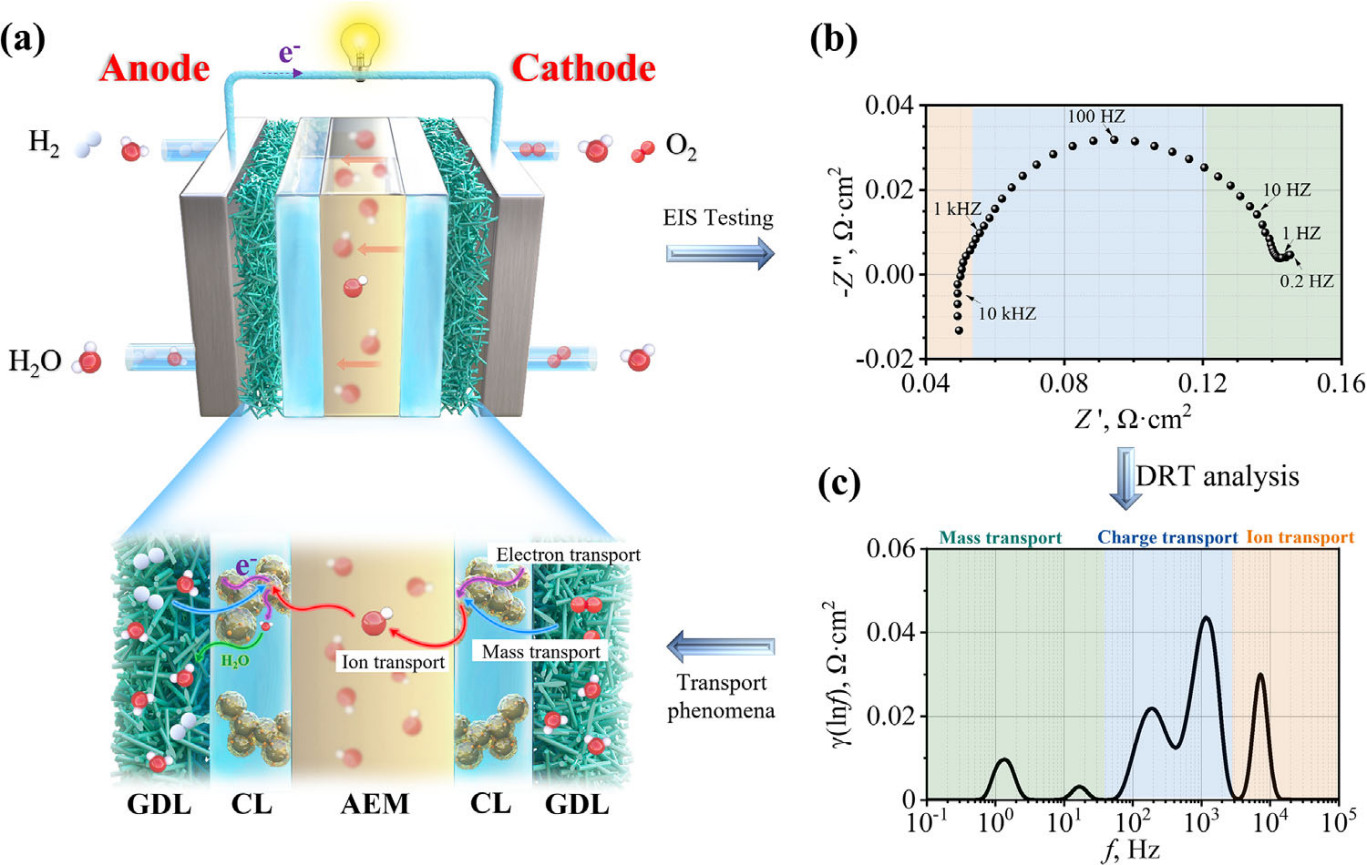
a) Schematic of AEMFCs, illustrating detailed transport processes of electrons, ions, gas, and water within the MEA; b) A typical Nyquist plot from EIS testing; c) and the corresponding DRT curve.

Recently, the model‐free approach of the distribution of relaxation times (DRT) has garnered increased attention as an alternative to ECM.^[^ ^29^, ^30^ ^]^ DRT does not require prior system knowledge and provides a method for decomposing impedance spectra based on relaxation times—the duration needed for a system to return to equilibrium after a perturbation. This method effectively deconvolutes overlapping processes in EIS data, making it particularly well‐suited for analyzing complex electrochemical systems such as fuel cells. In these systems, relaxation times correspond to specific time constants, with shorter relaxation times indicating faster transitions to the steady state. By resolving the relaxation times of different polarization processes, DRT enables the identification and separation of transport phenomena and electrochemical reaction processes within AEMFCs. For example, Eva Sediva et al.^[^ ^31^ ^]^ employed DRT to identify limiting loss processes in AEMFCs, revealing that the relative humidity of the inlet gas slightly affects DRT spectra at low current densities. Kangwei Qiao et al.^[^ ^32^ ^]^ observed that charge transfer resistance is the primary rate‐limiting step at high humidity and large current densities. Despite these advances, applying DRT analysis to diagnose AEMFC health states, particularly those related to water content, remains unexplored. In this study, we apply DRT to analyze impedance spectra under well‐defined experimental conditions, including membrane thickness, precious metal catalyst loading, inlet gas mass flow rates, and current density, to identify internal fuel cell dynamic processes based on their characteristic time constants (Figure 1). We further investigate the association between peak areas in DRT spectra and cell polarization losses under water flooding and drying conditions at the anode and cathode. Based on these insights, the degradation mechanisms of AEMFCs under three typical water content conditions—normal, cathode drying, and anode flooding—are examined. Analyzing impedance changes during durability tests clarifies the relationship between cell performance degradation and increased resistance in ion, charge, and mass transport.

## 2 Results and Discussion

### 2.1 Parameter Selection of EIS and DRT

**Figure 2:**
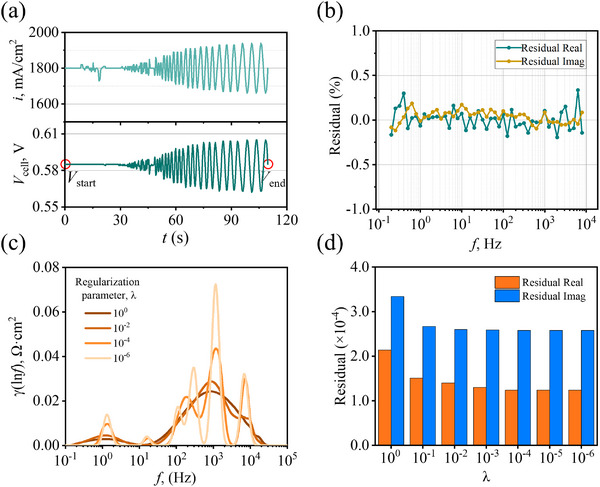
a) The relationship *i* and *V*_cell_ as a function of *t* during the EIS testing, where cell voltage variation is solely caused by the current perturbation; b) Computational relative residuals of the KK transformation as a function of *f*, with the real and imaginary components represented by green and yellow lines, respectively; c) DRT functions for variation of *λ* from 1 to 10^−6^; d) Corresponding DRT residuals as a function of *λ*, with the real and imaginary components represented by orange and blue bar, respectively.

EIS involves applying an AC perturbation of voltage (current) over a broad range of frequencies and monitoring the resulting variations in the magnitude and phase of the cell current (voltage) to determine the complex impedance of the AEMFCs under investigation. When a sinusoidal perturbation is applied to a linear system, the system's response signal is also sinusoidal and shares the same frequency as the applied perturbation. To maintain linearity in AEMFCs, the amplitude of the applied perturbation must be small. However, small perturbation amplitudes can result in a low signal‐to‐noise ratio. In this study, a perturbation amplitude of 5% of the AC current in galvanostatic EIS measurements was used to balance maintaining cell linearity with achieving a sufficient signal‐to‐noise ratio. To assess system stability during the EIS test, the voltage change rate (*δ*) was introduced.

(1) $$\delta =\frac{V_{\mathrm{start}}\;-\;V_{\mathrm{end}}}{V_{\mathrm{start}}}$$

where *V*_start_ and *V*_end_ represent the cell voltages at the start and end of the EIS test, respectively. **Figure** **2**a demonstrates the cell voltage variation under a current density of 1800 mA cm^−2^ with high stability (*δ* = 0.16%). Prior to analyzing the EIS data of AEMFCs using DRT, the Kramers‐Kronig (KK) transformation was applied to each EIS dataset to ensure high data quality.^[^ ^33^, ^34^ ^]^ Results indicate that the error remains below 1.0% across the entire measured frequency range (see Figure 2b), confirming the accuracy of the impedance measurements. Apart from the raw impedance data quality, the interpretability of DRT relies significantly on appropriate regularization during the calculation process. Figure 2c illustrates the substantial effect of the regularization parameter (*λ*) on the number and shape of identifiable peaks. At *λ* = 1, DRT analysis yields an overly smooth solution, identifying only two peaks. As *λ* decreases, the number of peaks rises and sharpens, heightening the risk of oscillations and over‐interpretation. A viable method to determine the optimal value for *λ* involves calculating residuals between measured impedance data and data reconstructed from DRT. Figure 2d shows that average real and imaginary residuals decrease as *λ* reduces from 1 to 10^−4^. Further reductions in λ have minimal impact on residuals but introduce additional peaks ≈ 110 Hz in the mid‐frequency region (see Figure 2c), leading to potential interference in data interpretation. Detailed residual distribution by frequency for different values of *λ* is shown in Figure S6 (Supporting Information). Considering the residuals and potential oscillatory errors, *λ* was set to 10^−4^ for subsequent DRT analysis.

### 2.2 Assignment of the DRT Function Peaks

**Figure 3:**
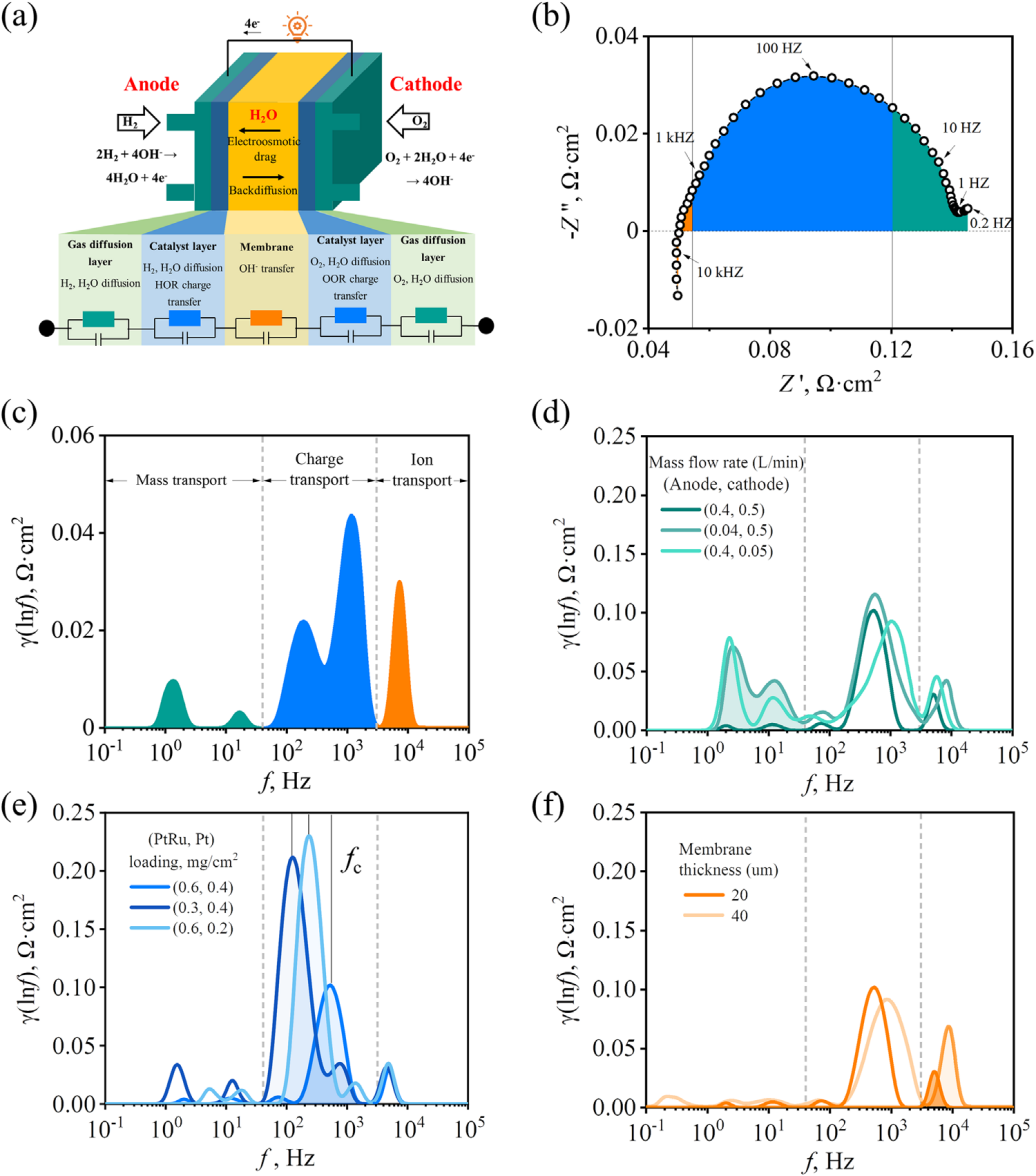
a) The ion, charge, and mass transport processes are illustrated at their respective locations in the AEMFC. b) Typical Nyquist plot at 1800 mA cm^−2^ under baseline MEA component and operating conditions; c) The corresponding DRT plot, divided into three regions, with ion, charge, and mass transport resistance represented by orange, blue, and green peak area, respectively; Employing the control variable method to adjust the MEA components and operating conditions relative to baseline: d) Inlet gas mass flow rate; e) Precious metal catalyst loading; f) Membrane thickness.

Impedance is analyzed using the DRT method, transforming the Nyquist plot from real and imaginary impedance into a resistivity versus frequency spectrum. This transformation enables the separation and quantification of polarization losses across various frequencies and timescales. Specific impedance for each polarization is obtained by integrating the corresponding DRT peaks, facilitating the quantification of polarization losses within the fuel cell. **Figure** **3**b illustrates a representative Nyquist plot at a current density of 1800 mA cm^−^^2^ under baseline MEA components and operating conditions, including a mass flow rate of 0.4 L min^−1^ for the anode and 0.5 L min^−1^ for the cathode, catalyst loading of 0.6 and 0.4 mg_Pt_ cm^−^^2^ for anode and cathode respectively, and a membrane thickness of 20 µm, covering a frequency range of 0.2 Hz to 20 kHz. An inductive effect appears in the high‐frequency range (greater than 7960 Hz) due to the measurement equipment and load wire. The real‐axis frequency intercept of the impedance spectra represents ohmic resistance, while the remaining part of the impedance relates to polarization resistance, the primary focus of DRT analysis. Figure 3c presents the DRT spectrum derived from EIS data, revealing five distinct peaks categorized into three regions. Each peak's area corresponds to area‐specific resistance, providing insights into the underlying dynamic processes. Identification of specific physicochemical processes associated with each DRT peak is crucial for operational diagnostics. To support this identification, MEA components and testing conditions are systematically adjusted to simulate variations in internal processes at *i* = 200 mA cm^−2^ (see Figure 3d–f). The relatively low current density ensures stable cell operation following adjustments across a wide range of operating conditions. To maintain high‐quality EIS results, relative residuals for both KK and *δ* transformations across all data are lower than 1% (see Figures S7 and S8, Supporting Information). The polarization curves for each operating condition are provided in Figure S9 (Supporting Information).

#### 2.2.1 Region I (0.2 – 40 Hz)

This region, containing two peaks, is attributed to mass transfer resistance. This attribution is supported by a strong correlation with changes in inlet reactant gas concentration (see Figure 3d) and a weak correlation with catalyst loading and membrane thickness. For instance, a notable increase in peak area within Region I is observed as H₂ or O₂ inlet concentrations decrease. Conversely, peak areas remain relatively unchanged when catalyst loading decreases (Figure 3e) or membrane thickness increases (Figure 3f). While Regions II and III show slight variations in peak areas with changing inlet gas concentrations, these changes are relatively minor and fall within experimental error. The integrated peak area of different regions is provided in Figure S10 (Supporting Information).

#### 2.2.2 Region II (40 – 3 kHz)

Encompassing two peaks, this region likely represents charge transfer resistance associated with the anodic hydrogen oxidation reaction (HOR) and the cathodic ORR. The catalyst layer composition has a significant influence on these processes. For example, reducing the anode precious metal catalyst loading from 0.6 to 0.3 mg_PtRu_ cm^−^^2^ significantly increases the peak area within this frequency range (see Figure 3e). Similarly, reducing the cathode precious metal catalyst loading notably increases the peak area in this region. Compared to PEMFCs, HOR kinetics in AEMFCs decrease, while ORR kinetics increase, leading to closer kinetics between both reactions. This makes it challenging to distinguish between HOR and ORR charge transfer resistance in the DRT spectra. Furthermore, as the precious metal catalyst loading decreases, the primary peak shifts to lower frequencies. When PtRu loading at the anode and Pt loading at the cathode decrease to 0.3 and 0.2 mg cm^−^^2^, respectively, the characteristic frequency (*f*_c_) in Region II reduces to 115 and 102 Hz, respectively. To account for peak overlap and frequency shifts, the frequency interval is divided, and the HOR and ORR charge transfer impedances are summed to improve the data's signal‐to‐noise ratio. For example, under operating conditions where HOR kinetics are weak (e.g., low current density), the HOR and ORR peaks may overlap significantly, and attempting to separate them could lead to data oscillation or artifacts.

#### 2.2.3 Region III (above 3 kHz)

This region encompasses a single peak representing ion migration resistance in both the electrode and membrane. As shown in Figure 3f, the peak area magnitude increases as membrane thickness rises from 20 to 40 µm, while other peaks remain largely unchanged.

### 2.3 Effect of Current Density

**Figure 4:**
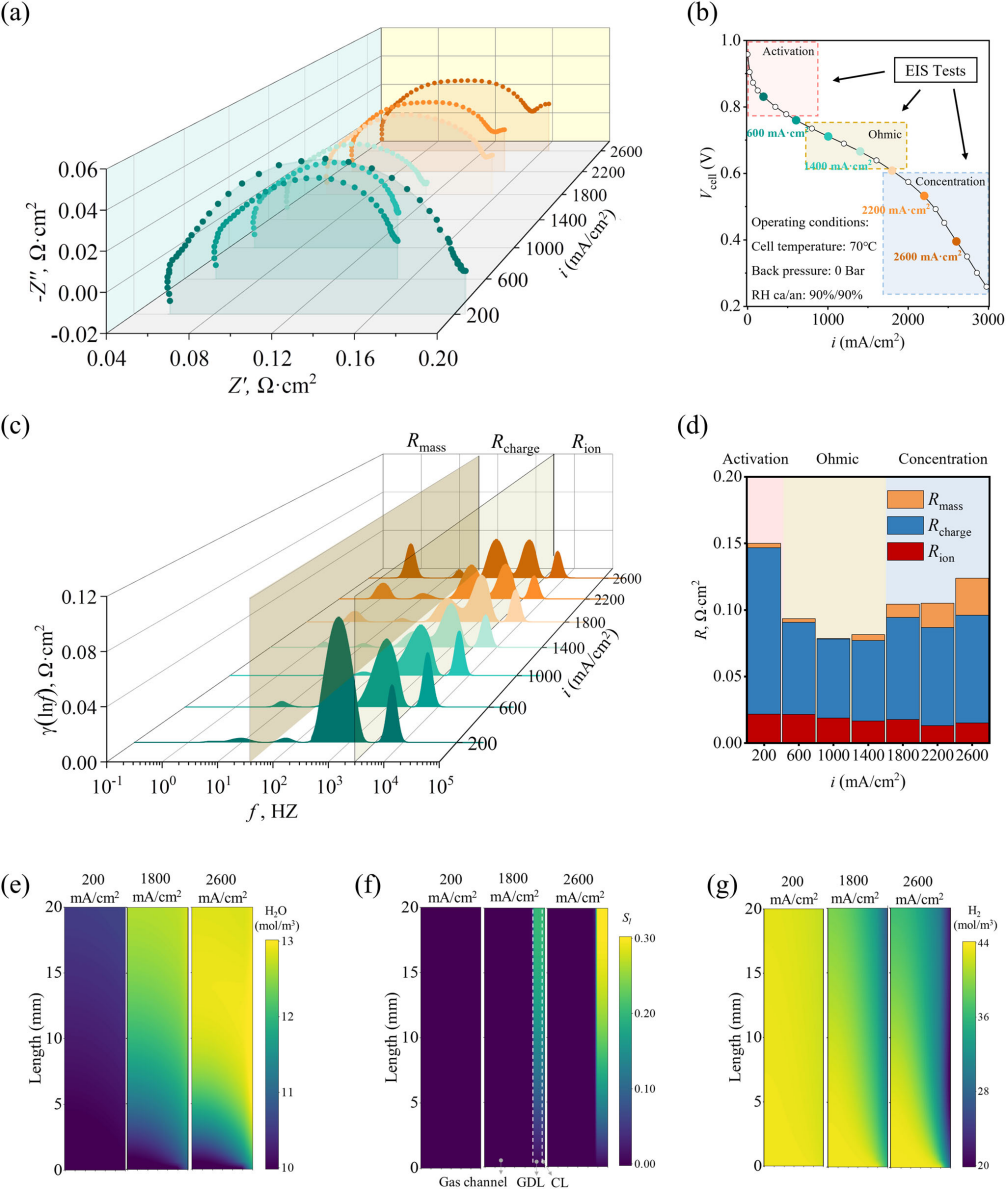
a) Impedance spectra across current densities ranging from 200 to 2600 mA cm^−^^2^ under the baseline operating conditions; b) Polarization curve of AEMFCs under the same baseline conditions, with the operating conditions depicted in the figure corresponding to the baseline setup; c) DRT plots as a function of current density; d) Integration of the DRT curves across different frequency ranges based on the spectra presented in (c), with ion, charge, and mass transfer resistances indicated by red, blue, and orange bars, respectively; e–g) Distributions of vapor concentration, liquid water volume fraction, and H_2_ concentration within the Anode Gas Channel, Anode GDL, and Anode CL domains for three representative current densities (200, 1800, and 2600 mA cm^−^^2^), respectively.

The steady‐state polarization curve reveals the characteristic relationship between electrode reaction rate and electrode potential. Understanding polarization characteristics of fuel cells—including ohmic, activation, and concentration polarization—requires impedance measurements across various current densities. EIS experiments are conducted across a range of current densities, from 200 to 2600 mA cm^−^^2^. As shown in **Figure** **4**a, Nyquist plots show an overlap of medium‐ and low‐frequency impedance arcs in the activation region (*i* < 600 mA cm^−^^2^). As current density increases to the ohmic region (600 mA cm^−^^2^ < *i* < 1800 mA cm^−^^2^), the distinction between medium‐ and low‐frequency arcs becomes more pronounced. At higher current densities, within the concentration region (*i* > 1800 mA cm^−^^2^), a tail with a 45° slope appears in the low‐frequency range. This indicates that varying equivalent circuit models are needed for different EIS data, leading to potential ambiguity in interpreting electrochemical processes. By contrast, DRT analysis, as a non‐parametric model, offers a more straightforward visualization of the impedance spectrum. Changes in the various impedance processes of the fuel cell are directly observable in the DRT spectrum. As shown in Figure 4c, impedance at various frequencies is distinguishable. With increasing current density, the peak area in the low‐frequency range gradually expands, indicating increased mass transport resistance. To quantify changes in transport resistance, impedance is obtained by integrating the peak area within specific frequency regions. Specifically, *R*_mass,_ *R*_charge_, and *R*_ion_ represent the transport resistances for mass, charge, and ions, respectively, and are calculated over the following frequency intervals: 0.2–40 Hz for *R*_mass_, 40–3000 Hz for *R*_charge_, and 3000–20,000 Hz for *R*_ion_.

In the low current density region, *R*_charge_ predominantly influences the overall reaction rate, decreasing rapidly as current density increases (see Figure 4d). For instance, *R*_charge_ decreases from 0.125 Ω·cm^2^ at 200 mA cm^−^^2^ to 0.691 Ω·cm^2^ at 600 mA cm^−^^2^. This trend is explained by the Butler‐Volmer equation, which indicates an inverse relationship between charge transfer resistance and current density. In the high current density region, *R*_charge_ experiences a slight increase due to elevated liquid water content, reducing available electrochemical reaction sites. As shown in Figure 4e,f, the average vapor concentration in the catalyst layer region increases from 10.331 to 12.966 mol m^−3^ with increasing current densities from 200 to 2600 mA cm^−^^2^, leading to a corresponding increase in the average volume fraction of liquid water (*S^l^*) from 0 to 0.21. These results corroborate the observed increase in *R*_mass_ in the DRT spectra (Figure 4b), as excess liquid water impedes H_2_ diffusion within the catalyst layer. This rise in vapor concentration results from two factors: first, greater water production at the anode via HOR as current density increases; second, intensified electroosmotic drag at higher current densities, driving water from cathode to anode. Meanwhile, the fluid velocity in the GDE is significantly lower than that in the gas channels, leading to the accumulation of liquid water within the electrode (see Figure S11, Supporting Information). This excess water causes electrode flooding, impeding H_2_ diffusion within the catalyst layer and increasing *R*_mass_ (see Figure 4g). Additionally, as current density increases, electrochemical reactions consume more water at the cathode, and intensified electromigration draws water from cathode to anode. This results in low water content in the cathode, further elevating mass transport resistance. However, increased water content at higher current densities also raises the hydration level of the membrane and electrolyte phase in the catalyst layer, reducing ionic transfer resistance. Therefore, *R*_ion_ decreases from 0.0218 Ω·cm^2^ at 200 mA cm^−^^2^ to 0.0152 Ω·cm^2^ at 2600 mA cm^−^^2^.

### 2.4 Effect of Relative Humidity

This section examines the effect of varying relative humidity (RH) on voltage loss and impedance to develop a water management fault detection scheme for AEMFCs. By adjusting the RH of the inlet gases, different water content conditions—flooding, normal, and drying—are simulated in the electrodes, and corresponding voltage and EIS measurements are recorded. The control variable method is used to alter RH selectively: when adjusting anode RH, cathode RH is maintained at 90%, and vice versa. These RH levels are chosen based on the optimal electrochemical performance of the AEMFC. Additionally, the working current density is set to 1800 mA cm^−^^2^ to enhance the water content difference between anode and cathode. This high current density amplifies water production at the anode and water consumption at the cathode. Moreover, the electroosmotic drag effect intensifies under these conditions, promoting water transfer from cathode to anode.

**Figure 5:**
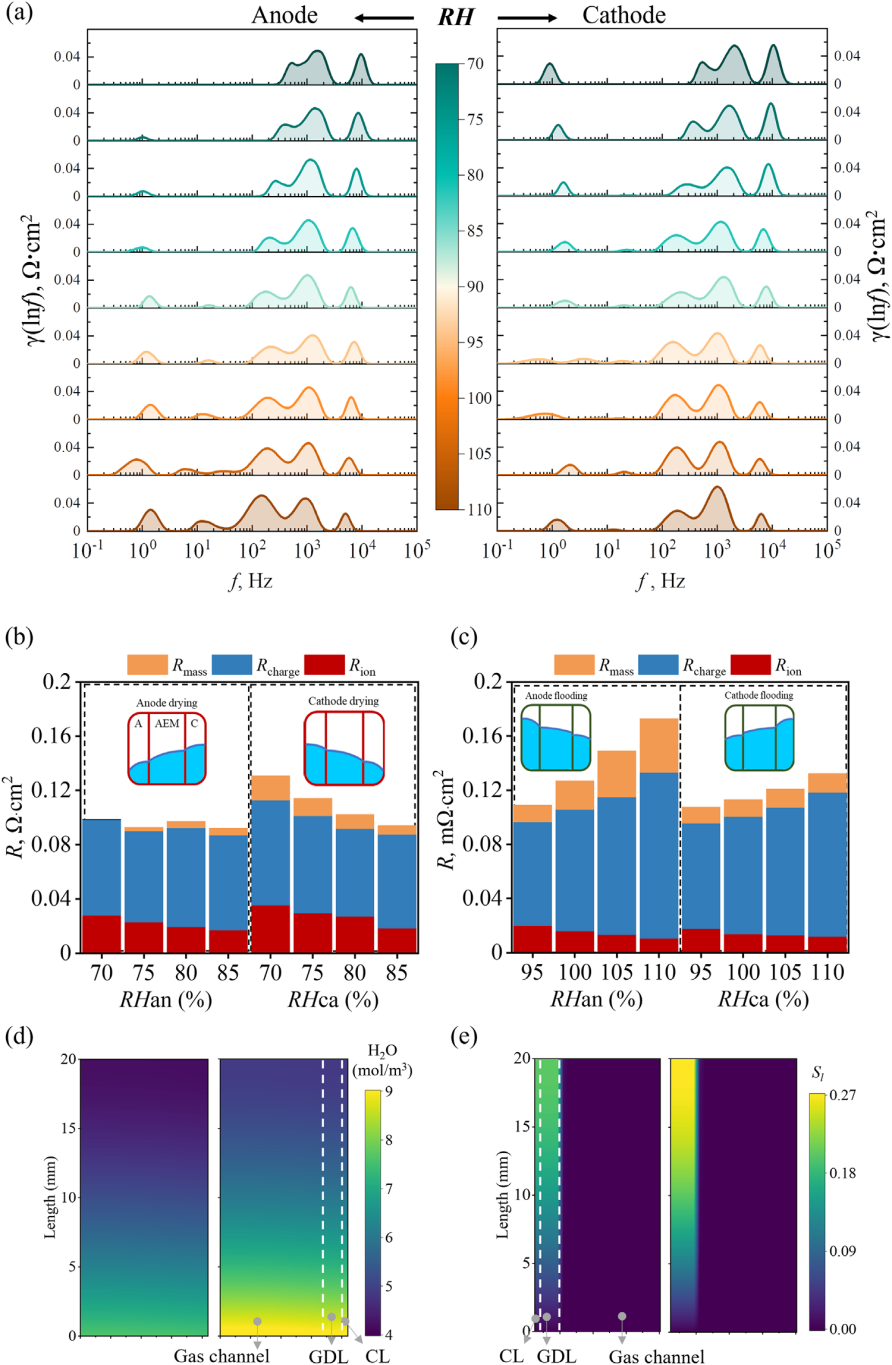
a) DRT curve as a function of RHan and RHca at the current density of 1800 mA cm^−2^. With baseline values for RHan and RHca set at 90%. Bar graphs show the integration results of DRT curves across different frequency ranges under two water management fault conditions: b) drying conditions; c) flooding conditions; d) vapor distribution on the cathode side for two representative RHca values (70% and 90%). e) *S_l_* distribution on the anode side for two representative typical RHan (90% and 110%).

Initially, reduced RH conditions are applied to simulate a drying scenario in the AEMFC. As shown in **Figure** **5**a, impedance in the high‐frequency region rises as RH decreases, resulting in significant cell voltage loss, particularly on the cathode side. This is primarily due to reduced water content, impairing membrane hydration, weakening ionic conductivity, and increasing cell resistance. For instance, *R*_ion_ decreases from 0.028 to 0.017 Ω·cm^2^ as anode relative humidity (RHan) rises from 70% to 85% (see Figure 5b). A more substantial increase in ionic transfer resistance occurs when cathode relative humidity (RHca) is reduced. Specifically, when RHca is lowered to 70%, *R*_ion_ increases to 0.036 Ω·cm^2^. Besides ionic transfer resistance, mass transport resistance also increases significantly with a reduction in RHca, as water is a critical reactant in the ORR. Reduced water content limits reaction kinetics. Figure 5d shows that the average water content in the cathode catalyst layer decreases from 6.30 mol m^−3^ at RHca = 85% to 5.37 mol m^−3^ at RHca = 70%, leading to an increase in *R*_mass_ from 0.0065 to 0.018 Ω·cm^2^. Therefore, distinguishing between anode and cathode drying is possible by analyzing changes in ionic transfer and mass transport resistance.

Under increased RH conditions, additional water is introduced into the AEMFC, enhancing membrane hydration and facilitating OH⁻ ion movement. Consequently, ionic transfer resistance decreases significantly with increasing RH at both the anode and cathode. For example, when RHan and RHca increase to 110%, ionic resistance decreases to 0.0107 and 0.012 Ω·cm^2^, respectively (see Figure 5c). In the medium‐frequency region, increasing RHan has a more pronounced effect on impedance than increasing RHca. Specifically, when RHan increases from 90% to 110%, *R*_charge_ increases by 61.8%, whereas when RHca increases from 90% to 110%, *R*_charge_ increases by only 33.5%. This effect is attributed to water condensation at the anode, which forms an increased liquid water film over the PtRu catalyst particles, thereby reducing the number of three‐phase boundary reaction sites. In contrast, charge transfer resistance increases only slightly with higher RHca, likely because the cathode ORR consumes water, and the cathode operates at a higher temperature, resulting in greater saturated vapor pressure (see Figure S12, Supporting Information). This causes a lower vapor concentration and reduced vapor liquefaction at the cathode, leaving mass transport resistance relatively unchanged with increasing RHca. However, mass transport resistance increases significantly with higher RHan. As discussed earlier, excess liquid water at the anode blocks pores in the porous electrode, hindering reactant gas diffusion. For example, the *S_l_* of the anode catalyst layer increases from 0.131 to 0.183 (see Figure 5e), causing mass transport impedance to rise from 0.0125 to 0.0399 Ω·cm^2^ as RHan increases from 90% to 110%. Therefore, distinguishing between anode and cathode flooding is possible by observing changes in ionic transfer and mass transport resistance.

### 2.5 Durability Tests

**Figure 6:**
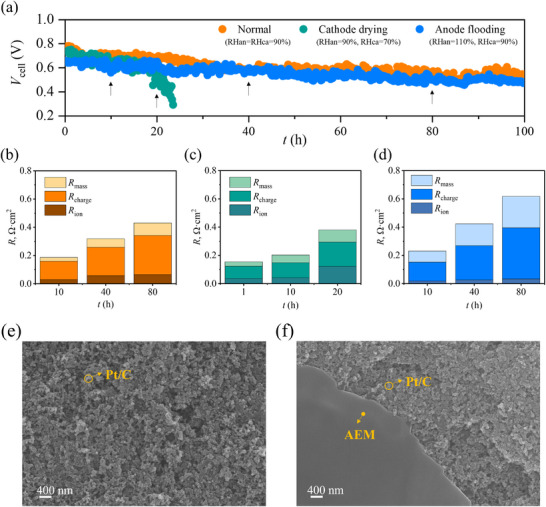
a) Cell voltage change of AEMFCs at 600 mA cm^−2^ over the short‐term durability test of 100 h, and upward arrows indicate the timing of EIS measurement. Bar graphs show the integration results of DRT curves across different frequency ranges under three water content conditions: b) Normal; c) Cathode drying; d) Anode flooding. Corresponding DRT plots is provided in Figure S13 (Supporting Information). The SEM image of the cathodic catalyst layer: e) before; f) after cathode drying conditions.

To further elucidate the impact of water management faults on AEMFC performance stability, a 100 h short‐term durability test is conducted at a constant current density of 600 mA cm^−^^2^, monitoring cell voltage over time to assess durability. The test is conducted under three typical water content conditions: normal, anode flooding, and cathode drying. Under normal operating conditions (RHan = RHca = 90%), as shown in **Figure** **6**a, voltage significantly decreases from 0.756 to 0.582 V within the first 40 h. This voltage drop is primarily attributed to membrane and ionomer degradation, reducing ionic transport performance. *R*_ion_ increases from 0.0288 Ω·cm^2^ at 10 h to 0.0656 Ω·cm^2^ at 40 h (see Figure 6b). As *t* increases from 40 to 80 h, further voltage decline is mainly due to deterioration of the catalyst layer microstructure leading to increased charge transfer impedance and mass transport impedance. Specifically, *R*_charge_ and *R*_mass_ increase to 0.276 and 0.0889 Ω·cm^2^, respectively, at *t* = 80 h.

Under cathode drying conditions (RHan = 90%, RHca = 70%), the initial voltage is 0.738 V, similar to that of the normal operating condition. However, under cathode drying conditions, the cell exhibits a more rapid voltage decline compared to normal operation with the cell voltage dropping to just 0.3 V after only 23 h of testing. This decline is attributed to the reduced RHca, which prevents the membrane and ionomer from absorbing sufficient water for optimal hydration. As a result, membrane and ionomer degradation accelerate, significantly increasing ionic transport impedance. *R*_ion_ increases substantially from 0.0367 Ω·cm^2^ at 1 h to 0.122 Ω·cm^2^ at 20 h (see Figure 6c). Prolonged exposure to low water content can lead to irreversible membrane and ionomer degradation, dramatically shortening the cell's operational lifespan. As shown in Figure 6e,f, a portion of the AEM membrane detached and adhered to the cathodic catalyst layer after the durability testing. This detachment may be attributed to prolonged over‐drying conditions, resulting in the chemical degradation of the AEM.^[^ ^7^ ^]^ This observation is consistent with the DRT findings (Figure 6c), which indicate a marked increase in *R*_ion_.

Under anode flooding conditions (RHan = 110%, RHca = 90%), the initial cell voltage of 0.652 V is the lowest among the three cases, likely due to increased *R*_charge_ and *R*_mass_. The excessive RH at the anode facilitates vapor condensation, resulting in liquid water that blocks electrode pores, reduces the electrochemically active surface area, and hinders mass transport. As shown in Figure 6d, *R*_charge_ and *R*_mass_ increase 2.71 and 2.83 times with increasing *t* from 10 to 80 h, respectively. However, this case exhibits the highest stability, in the first 40 h of durability testing, with cell voltage decreasing by only 0.081 V, corresponding to a degradation rate of 2.03 mV h^−1^. In comparison, the voltage decline under normal operating conditions is 0.174 V, with degradation rates of 4.35 mV h^−1^. This stability is primarily due to the higher RH, which improves membrane and ionomer hydration, promotes efficient OH^−^ transport, and mitigates chemical degradation. Consequently, *R*_ion_ only slightly increases from 0.0189 Ω cm^−^^2^ at 10 h to 0.0334 Ω cm^−^^2^ at 40 h (see Figure 6d).

## 3 Conclusion

This study has demonstrated a powerful methodology for diagnosing water management faults in AEMFCs using EIS testing and DRT analysis. DRT analysis enables the separation of multiple processes that are challenging to differentiate in the Nyquist plot. By adjusting MEA components and working conditions, three characteristic DRT regions are identified across different frequency ranges: mass transfer (0.2 – 40 Hz), charge transfer (40 – 30 Hz), and ion transfer (above 30 Hz). Furthermore, varying the current density clarifies the relationship between voltage loss and each polarization impedance. The limiting factor in MEA reaction kinetics shifts from ion transfer resistance to mass transfer resistance as current density increases. The relationship between each transfer resistance and water content is established by testing electrochemical impedance under various inlet gas relative humidity conditions. Using these insights, the type of water content failure (flooding or drying) occurring at the anode or cathode can be identified by analyzing changes in the DRT spectrum. Building on these findings, EIS‐DRT analysis is used to investigate degradation mechanisms in AEMFCs under three typical water content conditions. Results indicate that cell performance decay is primarily influenced by ion transport resistance, with the fastest decay occurring under cathode drying conditions and the slowest under anode flooding conditions. These findings highlight the effectiveness of using the EIS‐DRT analysis method for monitoring water management faults in AEMFCs. The insight gained from this method provides crucial data to support the implementation of water management control strategies, paving the way for enhanced performance and extended lifespan of commercial AEMFC stacks.

## 4 Experimental Section

### AEMFC Preparation—MEA Materials

The membrane electrode assembly (MEA) was fabricated using a 20 µm‐thick anion exchange membrane provided by Versogen (baseline case). The gas diffusion layer (GDL) on the anode side was Toray TPGH‐060, while Freudenberg H23C8 was used on the cathode side. The anode electrocatalyst comprised Pt‐Ru supported on high‐surface‐area carbon (SciMater‐DT), with a composition of 40 wt.% Pt and 20 wt.% Ru. For the cathode, Pt supported on Vulcan carbon (TEC10EA50E, Tanaka) was employed, containing 50 wt.% Pt.

### AEMFC Preparation—MEA Preparation

The catalyst ink was prepared by mixing the catalyst, an ionomer dispersion (5 wt.%, PiperION‐A5‐HCO3‐EtOH), and a solution of deionized water and isopropyl alcohol at a 1:2 weight ratio. The ionomer content in the ink was adjusted to 10 wt.% of the total solid content. The ink was then dispersed using an ultrasonic processor (Supmile, KQ‐300DE) for 1 h. Subsequently, the ink was uniformly sprayed onto the GDL using an ultrasonic spray system (Sonotek), forming a gas diffusion electrode (GDE) with an effective area of 4 cm^2^. For the baseline case, catalyst loadings were 0.6 mg_PtRu_ cm^−^^2^ for the anode and 0.4 mg_Pt_ cm^−^^2^ for the cathode.

### AEMFC Preparation—Single‐Cell Assembly

Before assembly, the membrane and GDE were pretreated in a 1.0 m KOH solution for 24 h to facilitate ion exchange. The single cell was then assembled using the pretreated MEA, two PTFE gaskets, graphite flow field plates, gold‐coated stainless steel current collectors, and end plates (see Figure S5, Supporting Information).

### AEMFC Test

All tests were conducted using the 850E Scribner Fuel Cell Test Station, which precisely controlled the cell temperature and inlet gas dew point. The cell temperature was maintained at 70 °C throughout the experiments. Before performance testing, the cell was conditioned at a constant voltage of 0.5 V, allowing the current density to increase gradually until reaching a steady state. For baseline cases, humidified hydrogen and oxygen were supplied to the anode and cathode at flow rates of 0.4 and 0.5 L min^−1^, respectively, with no backpressure applied. The inlet gas relative humidity (RH) for the anode and cathode was 90%. EIS measurements were performed in galvanostatic mode, with a frequency range of 0.2 Hz to 20 kHz and an AC signal amplitude set at 5% of the DC current.

### DRT Analysis

The reliability of the original EIS data is essential, as low‐quality data can result in DRT spectra that lack physical significance and are therefore unsuitable for interpreting electrochemical systems. To ensure data reliability, the Kramers‐Kronig (KK) validity test was applied to all EIS datasets in this study. Datasets with a relative residual below 1% were considered valid for further analysis and conversion to DRT. In DRT analysis, the impedance spectra were modeled using a series of RC elements, allowing each electrochemical process to be distinguished based on its intrinsic time constant. This approach generates a distribution of impedance as a function of the time constants (*τ*  =  *RC*). The relationship between the distribution function, γ(*τ*), and the cell impedance, *Z*_cell_(*f*), is described by

(2) $$Z_{\mathrm{cell}}\left(f\right)=R_{0}+Z_{\mathrm{pol}}\;\left(f\right)=R_{0}+\int_{-\infty }^{\infty }\frac{\gamma (\ln\tau )}{1+j2\pi f\tau }d\ln\tau$$

where *f* is the frequency of excitation, *R*_0_ is the high–frequency cell resistance, *Z*_pol_(*f*) is the polarization impedance, *j* is the imaginary quantity. The detailed derivation process of DRT analysis is detailed in Section S1 (Supporting Information). The DRT transformation of EIS spectra was run in the MATLAB GUI toolbox created by Ciucci's research group.^[^ ^35^ ^]^ To further understand the charge and mass transport of AEMFCs, a 2D Multiphysics model accounting for heat and mass transfer, fluid flow, multi‐phase water transport, and electrochemical reactions was developed. A detailed description of the model, including the governing equation and boundary conditions, is provided in Section S2 (Supporting Information).

## Conflict of Interest

The authors declare no conflict of interest.

## Supporting information



Supporting Information

## Data Availability

The data that support the findings of this study are available from the corresponding author upon reasonable request.
