# Supplementary material for: Water Management Fault Diagnosis by Operando Distribution of Relaxation Times Analysis for Anion Exchange Membrane Fuel Cells
Source: Adv Sci (Weinh). 2025 May 14;12(28):2505304. doi: 10.1002/advs.202505304 (PMC12302547; doi:10.1002/advs.202505304)
Supplement: Supplementary file 1 — Supporting Information [file ADVS-12-2505304-s001.docx]

**Water Management Fault Diagnosis by Operando Distribution of Relaxation Times Analysis for Anion Exchange Membrane Fuel Cells**

Haodong Huang^1, 2^, Zijie Zhang^1, 2^, Cailin Xiao^1, 2^, Jiapeng Liu^3^, Zheng Li^1, 2^, Yuting Jiang^1, 2^, Lei Wei^1, 2^, Tianshou Zhao^1, 2^*, Francesco Ciucci^4, 5*^, Lin Zeng^1, 2^*

^1^Shenzhen Key Laboratory of Advanced Energy Storage, Department of Mechanical and Energy Engineering, Southern University of Science and Technology, Shenzhen 518055, China

^2^SUSTech Energy Institute for Carbon Neutrality, Southern University of Science and Technology, Shenzhen 518055, China

^3^School of Advanced Energy, Sun Yat-Sen University, Shenzhen, China, 518107

^4^University of Bayreuth, Chair of Electrode Design for Electrochemical Energy Systems, Weiherstraße 26, 95448 Bayreuth, Germany

^5^University of Bayreuth, Bavarian Center for Battery Technology (BayBatt), Universitätsstraße 30, 95447 Bayreuth, Germany

*Corresponding Author: [zhaots@sustech.edu.cn](mailto:zhaots@sustech.edu.cn) (Tianshou Zhao), [Francesco.ciucci@uni-bayreuth.de](mailto:Francesco.ciucci@uni-bayreuth.de) (Francesco Ciucci), [zengl3@sustech.edu.cn](mailto:zengl3@sustech.edu.cn) (Lin Zeng)

# S1 DRT analysis

In DRT analysis, the relationship between the distribution function $\text{γ}\text{(ln}\text{τ}\text{)}$ and the polarization impedance $\text{Z}_{\text{pol}}\left( \text{f} \right)$ is described by:

$\text{Z}_{\text{pol}}\left( \text{f} \right)=\int_{\text{-∞}}^{\text{∞}} \frac{\text{γ}\text{(ln}\text{τ}\text{)}}{\text{1 + }\text{j}\text{2π}\text{fτ}}\text{d}\text{ln}\text{τ}$ (S1)

The expression represents the first-kind Fredholm integral equation, in which $\text{γ}\text{(ln}\text{τ}\text{)}$ is an infinite-dimensional continuous function. Due to the inherent difficulty in solving such equations analytically, numerical techniques are typically employed. The $\text{γ}\text{(ln}\text{τ}\text{)}$ is discretized using a set of basis function $\text{ϕ}_{\text{m}}\text{(ln}\text{τ}\text{)}$, yielding:

$\text{ }\text{ }\text{γ}\text{(ln}\text{τ}\text{)}\text{ }\text{=}\text{ }\sum_{\text{m}\text{=1}}^{\text{M}} \text{x}_{\text{m}}\text{ϕ}_{\text{m}}\text{(ln}\text{τ}\text{)}\text{+}\text{e}^{\text{discr}}\text{(ln}\text{τ}\text{)}$ (S2)

where $\text{x}_{\text{m}}$ denotes the weighting coefficient associated with each time constant $\text{τ}_{\text{m}}$ and $\text{e}^{\text{discr}}$ represents the discretization error. Substituting Eq.(S2) into Eq.(S1) and rearranging into matrix form gives:

$\text{Z}_{\text{cell}}\left( \text{f} \right)\text{ }\text{= }\text{R}_{\text{0}}\text{I}\text{ }\text{+}\text{ }\text{A}^{\text{'}}\text{x}\text{ }\text{+}\text{ }\text{i}\text{A}^{\text{''}}\text{x}\text{ }\text{+}\text{ }\text{e}^{\text{discr}}$ (S3)

where $\text{Z}_{\text{cell}}\left( \text{f} \right)$ is the modeled complex impedance vector of size $\text{N × 1}$, $\text{x}$ is the vector of unknown coefficients (of dimension M × 1). $\text{I}$ is an N × 1 unit vector, $\text{A}^{\text{'}}$and $\text{A}^{\text{''}}$ are N × M matrices corresponding to the real and imaginary components, respectively. The DRT analysis then aims to determine $\text{x}$ by solving the following constrained least-squares optimization problem:

$\text{x}\text{ }\text{=}\text{ }\underset{\text{x}\text{ }\text{>}\text{ }\text{0}}{\text{argmin}} \text{[}\left\| \text{Ω}^{\text{'}}\left( \text{Z}_{\text{real}} \text{-}\text{ }\text{R}_{\text{0}}\text{I}\text{ }\text{-}{\text{ }\text{A}}^{\text{'}}\text{x} \right) \right\|_{\text{2}}^{\text{2}}\text{ }\text{+}\left\| \text{Ω}^{\text{''}}\text{(}\text{Z}_{\text{im}} \text{-}\text{ }\text{A}^{\text{''}}\text{x}\text{)} \right\|_{\text{2}}^{\text{2}}\text{]}$ (S4)

where $\text{Ω}^{\text{'}}$ and Ω′′\Omega''Ω′′ are diagonal weighting matrices. Given that ${\text{ }\text{A}}^{\text{'}}$ and $\text{A}^{\text{''}}$ may be ill-conditioned, directly solving Eq. (S4) can lead to non-unique or unstable solutions, often sensitive to measurement noise and prone to overfitting. To alleviate these issues, a regularization term is incorporated into the objective function:

$\text{x}\text{ }\text{=}\underset{\text{x}\text{ }\text{>}\text{ }\text{0}}{\text{argmin}} \text{[}\left\| \text{Ω}^{\text{'}}\left( \text{Z}_{\text{real}} \text{-}\text{ }\text{R}_{\text{0}}\text{I}\text{ }\text{-}{\text{ }\text{A}}^{\text{'}}\text{x} \right) \right\|_{\text{2}}^{\text{2}}\text{ }\text{+}\left\| \text{Ω}^{\text{''}}\text{(}\text{Z}_{\text{im}} \text{-}\text{ }\text{A}^{\text{''}}\text{x}\text{)} \right\|_{\text{2}}^{\text{2}}\text{ }\text{+}\text{ }\text{λ}\left\| \text{Lx} \right\|_{\text{2}}^{\text{2}}\text{]}$ (S5)

In this formulation, $\text{L}$ is a discrete differential operator (e.g., first- or second-order) that enforces solution smoothness, and *λ* is the regularization parameter that balances data fidelity with numerical stability.

# S2 AEMFCs numerical model

A two-dimensional multi-physics model for AEMFCs was developed, incorporating water phase transitions, heat and mass transfer, liquid and dissolved water transport, and electrochemical reactions. The model employed a two-phase flow approach to capture the transport of both vapor and liquid water in the gas diffusion and catalyst layers. It considered three water phases—liquid, vapor, and dissolved.

## S2.1 Governing equations.

**Charge Transport:** The model accounts for the transport of two charged species: electrons and hydroxide ions, between the anode and cathode in an AEMFC. Electrons move through solid conductive materials (including the catalyst layers, microporous layers, and gas diffusion layers), generating electronic current, while hydroxide ions travel through the membrane and ionomer, resulting in ionic current.

**Heat Transfer:** Assuming thermal equilibrium across all phases, heat transfer within AEMFCs involves both conduction and convection. The model incorporates various heat sources, including those generated by electrochemical reactions, Joule heating (ohmic heating), and water phase transitions.

**Fluid Flow:** The gases at both the anode and cathode are modeled as ideal gases and are transported via diffusion through the gas channels, gas diffusion layers, and catalyst layers. The membrane is assumed to be impermeable to hydrogen, oxygen, and nitrogen. Momentum conservation is described using the Navier-Stokes equations in vector form, with the Darcy term applied specifically to porous media.

**Dissolved Water Transport:** The transport of dissolved water within the membrane and ionomer phases of the catalyst layer is characterized by the Nernst-Planck equation, which integrates diffusion and electro-osmotic drag.

**Gas Transport:** Gas transport is driven by both diffusion (concentration gradients) and convection (pressure gradients). The Fick’s diffusion model is employed to capture gas diffusion within the gas channels, porous electrode.

**Liquid Water Transport:** Liquid water transport is modeled using a volume-averaged approach to the continuity equation, combined with Darcy’s law to describe the movement of liquid water. The transport is primarily driven by the pressure differential between the gas and liquid phases (capillary pressure). Liquid water exists only in the anode and is generated by vapor condensation and desorption from the membrane/ionomer.

## S2.2 Boundary conditions

The boundary conditions applied in the AEMFC model were as follows:

1. The gas pressure at the outlet was set equal to atmospheric pressure, while the inlet reactant gas velocity was defined as *U*_in_.
2. At the inlet boundary, the mass fraction of each gas component was specified. At the outlet surfaces, gas transport was assumed to occur solely through convection.
3. The interface between the anode gas channel and the anode gas diffusion layer was grounded, while the interface between the cathode gas channel and the cathode gas diffusion layer was set to the working voltage (V_cell_).
4. A constant temperature boundary condition was imposed on the outer surface of the gas channels.
5. The inlet boundary condition of the gas channel included a specified volume fraction of liquid water, calculated based on the relative humidity of the inlet gas.


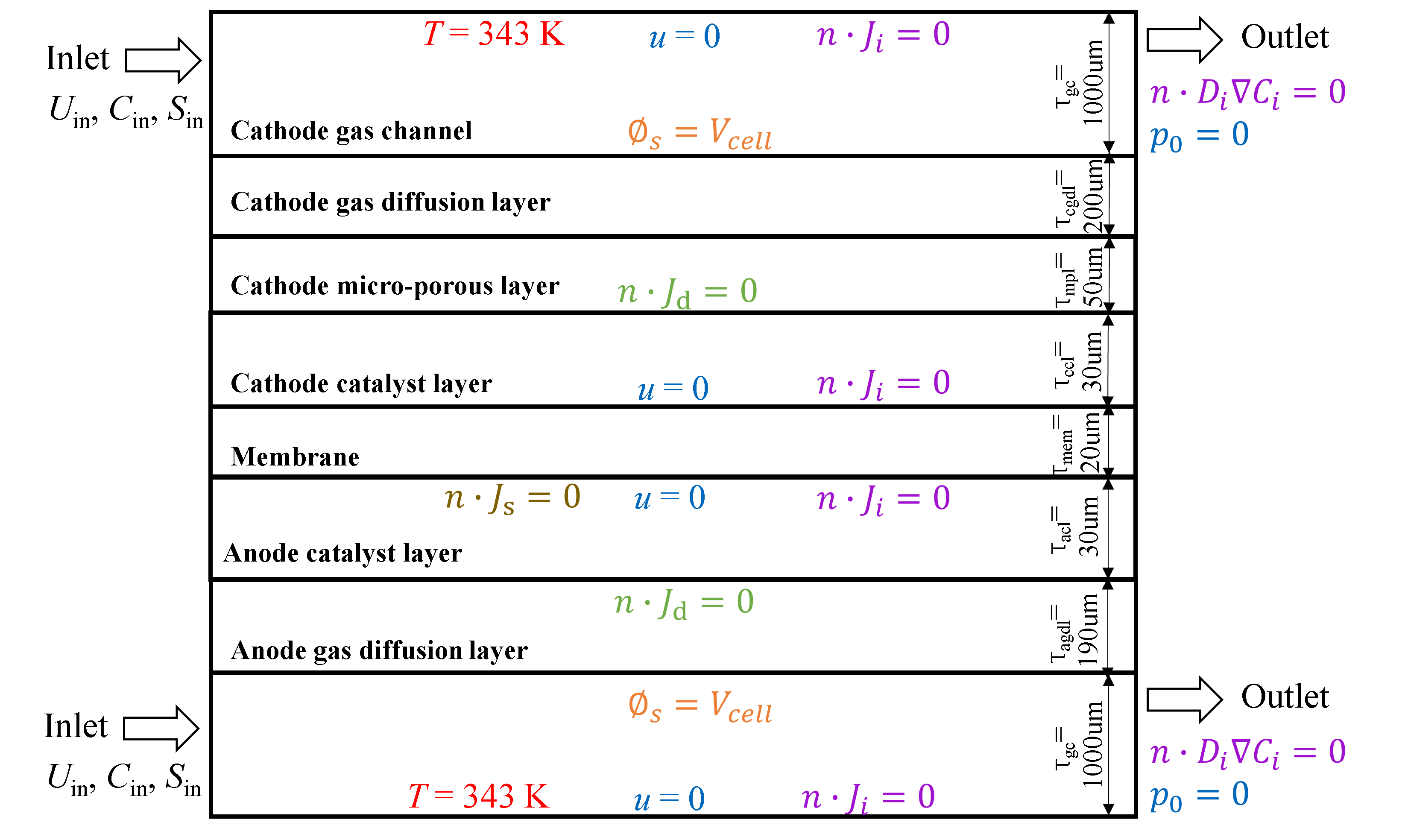


Fig. S1 The boundary conditions and geometry parameters of AEMFCs simulation model

## S2.3 Solver setting

In this study, the finite element method is employed to solve a fully coupled multiphysics model of the AEMFCs. Nonlinear equations are handled using Newton’s method, while the associated linear systems are solved with the parallel direct sparse solver (PARDISO), ensuring both numerical robustness and computational efficiency in addressing the complex coupling among physical fields. All simulations are performed using the commercial software COMSOL Multiphysics. The overall modeling workflow is illustrated in Fig. S2. Key boundary conditions are first defined, including the operating voltage, gas-phase relative humidity, flow rates and species concentrations, as well as the liquid water volume fraction. The model simultaneously resolves six interdependent physicochemical processes: electrochemical reactions, liquid water transport, dissolved water diffusion, gas-phase reactant transport, fluid dynamics, and heat transfer. These processes are tightly coupled throughout the simulation through bidirectional interactions involving shared variables and source terms, enabling a fully integrated and self-consistent resolution of the fuel cell’s multiphase transport and electrochemical behavior. A relative tolerance of 10^-4^ is applied to all governing equations to ensure numerical convergence and the reliability of the simulation outcomes.


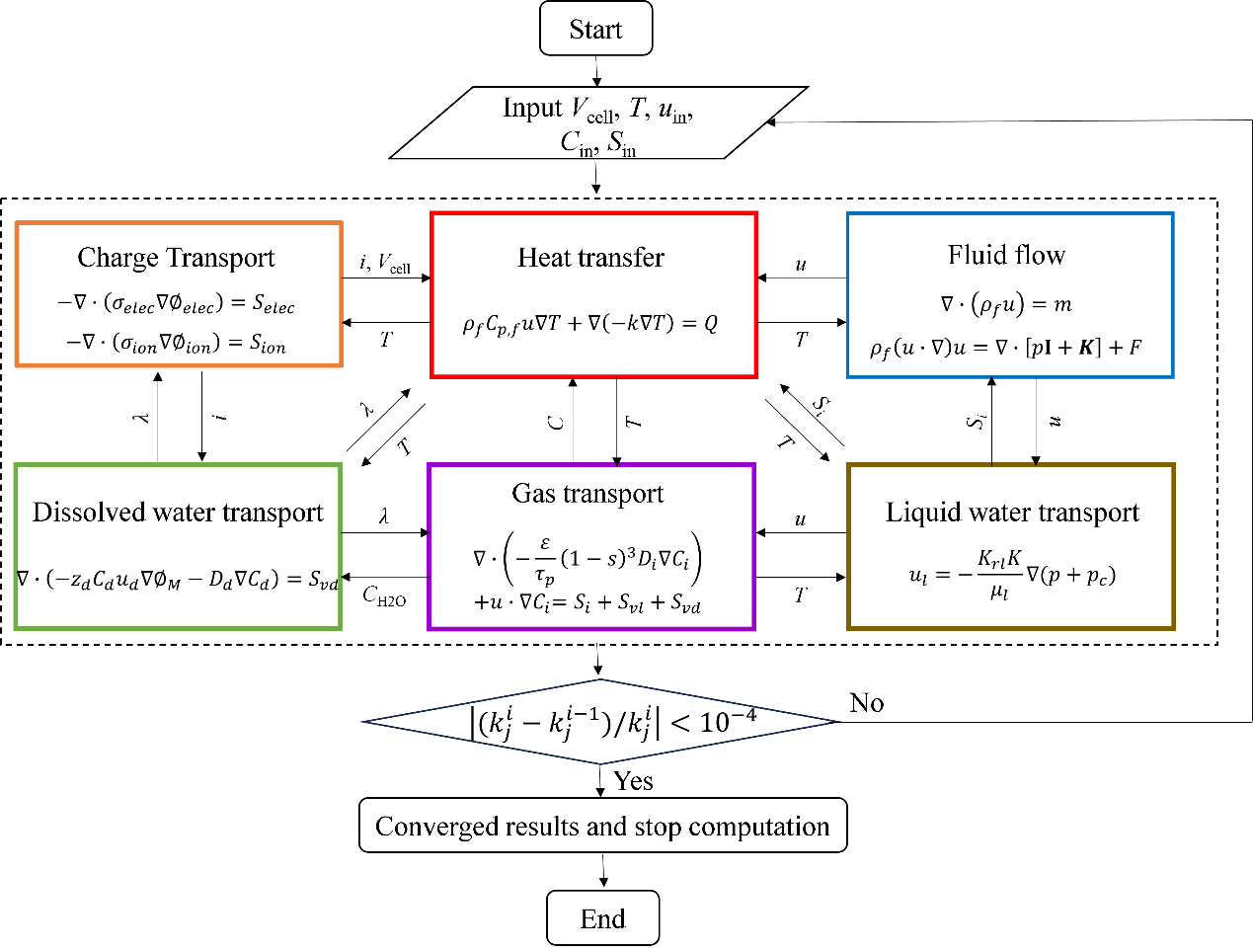


Fig. S2 The governing equations and work flowchart of AEMFCs simulation model

## S2.4 Physical property parameters

| Parameter | Nomenclature | Value | Unit |
| --- | --- | --- | --- |
| H_2_ diffusivity | $\text{D}_{\text{H}_{\text{2}}}$ | $\text{1.06×}\text{10}^{\text{-4}}\left( \frac{\text{T}}{\text{333.15}} \right)^{\text{1.5}}\text{(}\frac{\text{101325}}{\text{p}}\text{)}$ | m^2^/s |
| O_2_ diffusivity | $\text{D}_{\text{O}_{\text{2}}}$ | $\text{2.65×}\text{10}^{\text{-4}}\left( \frac{\text{T}}{\text{333.15}} \right)^{\text{1.5}}\text{(}\frac{\text{101325}}{\text{p}}\text{)}$ | m^2^/s |
| Vapor diffusivity | $\text{D}_{\text{a}\text{,} \text{H}_{\text{2}}\text{O}}$ | $\text{1.06}\text{×}\text{10}^{\text{-4}}\left( \frac{\text{T}}{\text{333.15}} \right)^{\text{1.5}}\text{(}\frac{\text{101325}}{\text{p}}\text{)}$ | m^2^/s |
| Dissolved water diffusivity | $\text{D}_{\text{d}}$ | $\text{4.14}\text{ }\text{×}{\text{ }\text{10}}^{\text{-8}}\text{λ}\left( \text{161}\text{e}^{\text{-}\text{λ}}\text{ }\text{+}\text{ }\text{1} \right)\text{e}^{\text{-}\frac{\text{2346}}{\text{T}}}$ | m^2^/s |
| Permeability | $\text{K}$ | $\frac{\text{d}_{\text{p}}^{\text{2}}}{\text{180}}\frac{\text{ε}^{\text{3}}}{{\text{(1-}\text{ε}\text{)}}^{\text{2}}}$ | m^2^ |
| Dynamic viscosity | $\text{μ}$ | -1.1×10^-7^+7.9×10^-8^*T*-7.2×10^-11^  *T* ^2^+5.2×10^-14^*T* ^3^-1.6×10^-17^*T* ^4^ | Pa∙s |
| Fluid density | $\text{ρ}_{\text{f}}$ | $\frac{\text{p}_{\text{ref}}\sum_{\text{i}} \text{x}_{\text{i}}\text{M}_{\text{i}}}{\text{R}\text{T}}$ | kg/m^3^ |
| CL density | $\text{ρ}_{\text{CL}}$ | 1000 | kg/m^3^ |
| GDL density | $\text{ρ}_{\text{GDL}}$ | 1000 | kg/m^3^ |
| MEM density | $\text{ρ}_{\text{MEM}}$ | 1980 | kg/m^3^ |
| Thermal conductivity | $\text{k}$ | 5 | W/m/K |
| H_2_ specific heat capacity | $\text{C}_{\text{p}\text{,}{\text{ }\text{H}}_{\text{2}}}$ | 1.91×10^-6^*T* ^2^-8.3×10^-4^*T*+28.89 | J/mol/K |
| O_2_ specific heat capacity | $\text{C}_{\text{p}\text{,}{\text{ }\text{O}}_{\text{2}}}$ | -4.28×10^-6^*T* ^2^+1.37×10^-2^*T*+25.43 | J/mol/K |
| N_2_ specific heat capacity | $\text{C}_{\text{p}\text{,}{\text{ }\text{N}}_{\text{2}}}$ | 1.79×10^-5^*T* ^2^+2.92×10^-3^*T*+27.85 | J/mol/K |
| Vapor specific heat capacity | $\text{C}_{\text{p}\text{,}\text{ }\text{v}}$ | 1.18×10^-6^*T* ^2^+9.62×10^-3^*T*+30.33 | J/mol/K |
| CL specific heat capacity | $\text{C}_{\text{p}\text{,}\text{ }\text{CL}}$ | 3300 | J/kg/K |
| MPL specific heat capacity | $\text{C}_{\text{p}\text{,}\text{ }\text{MPL}}$ | 3300 | J/kg/K |
| MEM specific heat capacity | $\text{C}_{\text{p}\text{,}\text{ }\text{MEM}}$ | 833 | J/kg/K |
| GDL specific heat capacity | $\text{C}_{\text{p}\text{,}\text{ }\text{GDL}}$ | 568 | J/kg/K |

**S2.5 Mesh independence analysis**

In finite element simulations, complex physicochemical problems are discretized by partitioning the computational domain into a large number of small mesh elements. Macroscopic conservation equations are solved within each element, and the resulted local solutions are assembled to yield the global solution. Consequently, mesh quality has a critical influence on both the accuracy and computational efficiency of the numerical model. To assess the suitability of the meshing strategy, a mesh independence study was conducted by examining the sensitivity of key physicochemical variables to variations in mesh density. As illustrated in Fig. S3, at a current density of 1800 mA/cm², the cell voltage initially decreases before reaching a plateau with increasing mesh refinement. Similarly, the volume fraction of liquid water and the temperature within the catalyst layer (CL) show an initial increase followed by a plateau as mesh refinement increases.When the mesh size reaches 7000 elements, variations in cell voltage, volume fraction of liquid water, and temperature are reduced to below 0.002 V, 0.001, and 0.5 K, respectively, indicating that the model has achieved the desired level of numerical accuracy. Although further refinement could marginally enhance precision, it would impose a considerable computational burden. Therefore, a mesh configuration comprising 7000 elements and 94,657 degrees of freedom was adopted to strike an optimal balance between accuracy and computational efficiency.


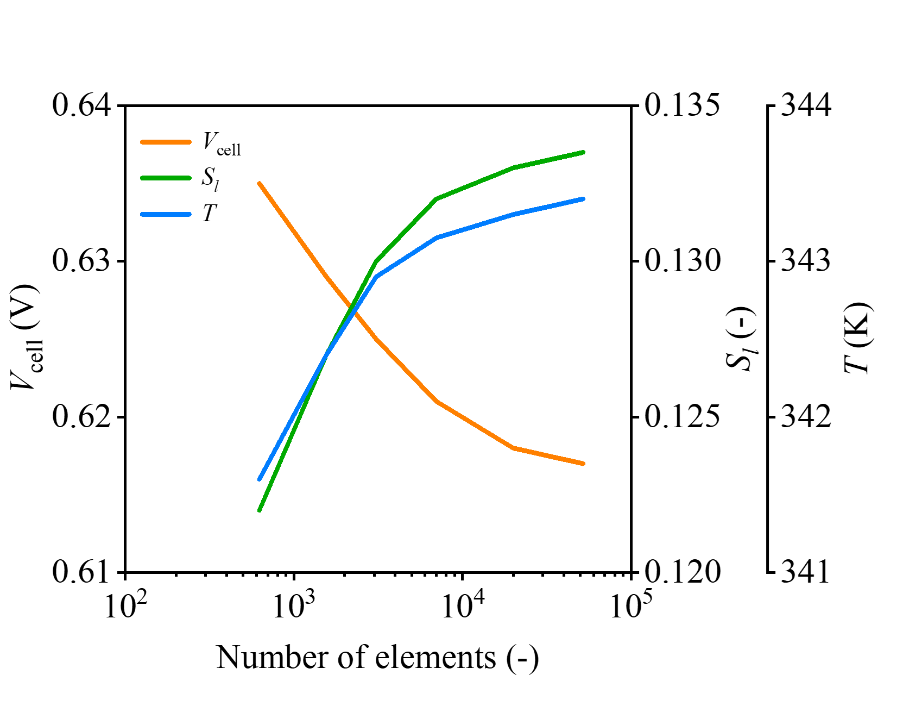


Fig. S3 Cell voltage,volume fraction of liquid water, and temperature as a function of number of mesh elements.

**S2.6 Experimental validation:**

Numerical models are generally based on assumptions and simplifications, often involving the idealization or omission of complex physicochemical phenomena. These approximations can inevitably introduce specific degrees of error, making it essential to validate the model’s accuracy through comparison with experimental data. In fuel cell research, polarization curves are widely employed for model validation, as they capture the nonlinear relationship between current density and output voltage. These curves encapsulate the effects of activation, ohmic, and concentration overpotentials, thereby offering valuable insights into the influence of multi-component transport processes on electrochemical reaction kinetics. As shown in Fig.S4, under the reference operating condition, the simulation results of the numerical model closely match the experimental measurements. For example, at a current density of 1800 mA/cm^2^, the experimentally measured voltage is 0.608 V, while the model predicts 0.621 V, corresponding to a relative error of only 2.13 %. These results demonstrate the model’s high predictive accuracy and capacity to reliably capture the system’s electrochemical behavior.


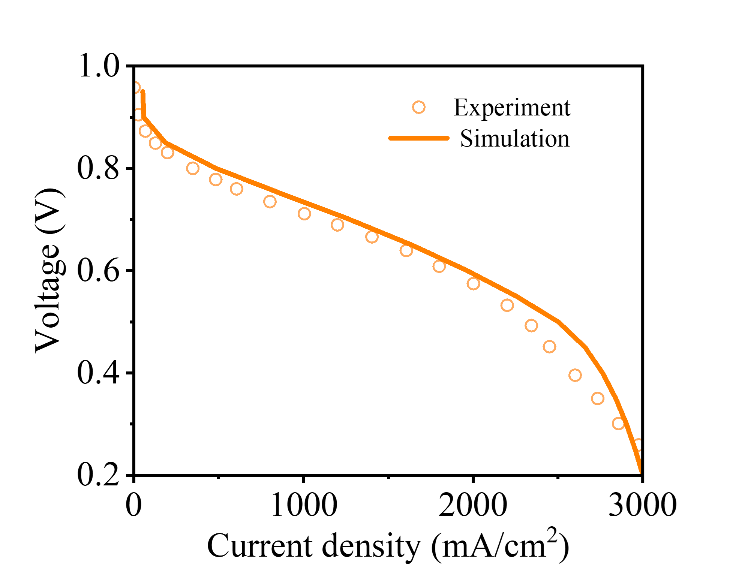


Fig. S4 Polarization curves for experimental tests and simulation results

## S2.7 Fuel cell test fixture


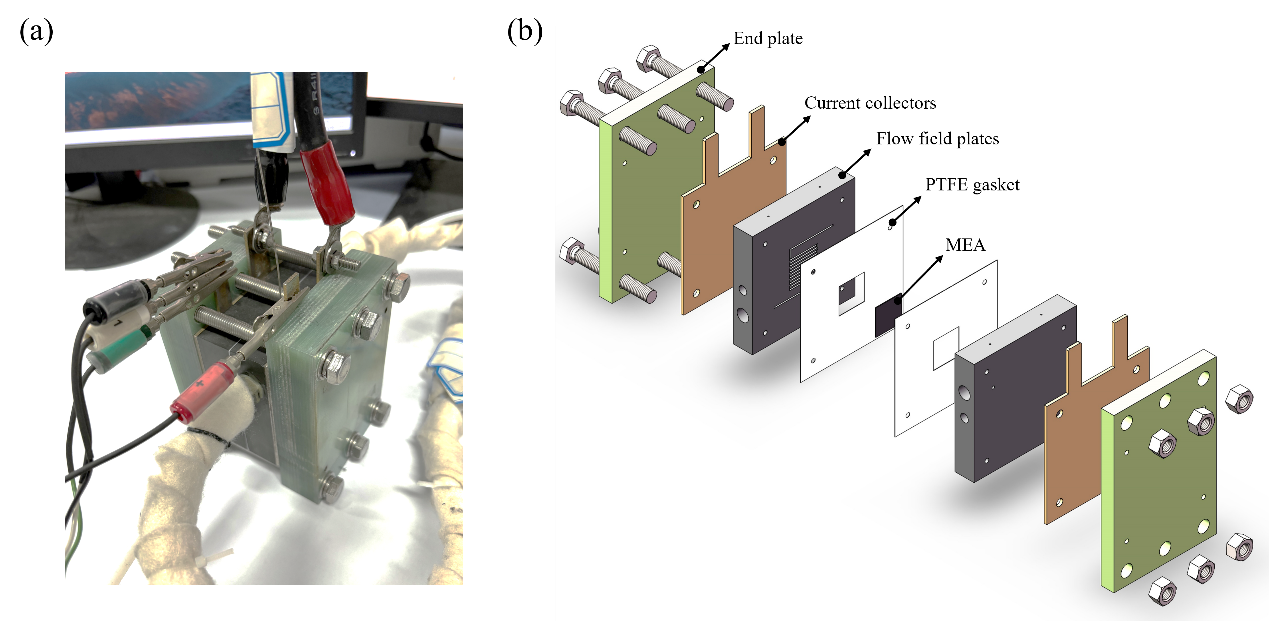
Fig. S5 (a) Single-cell fuel cell test fixture; (b) Schematic diagram of single-cell test cell components

# S3 Parameter selection of EIS and DRT


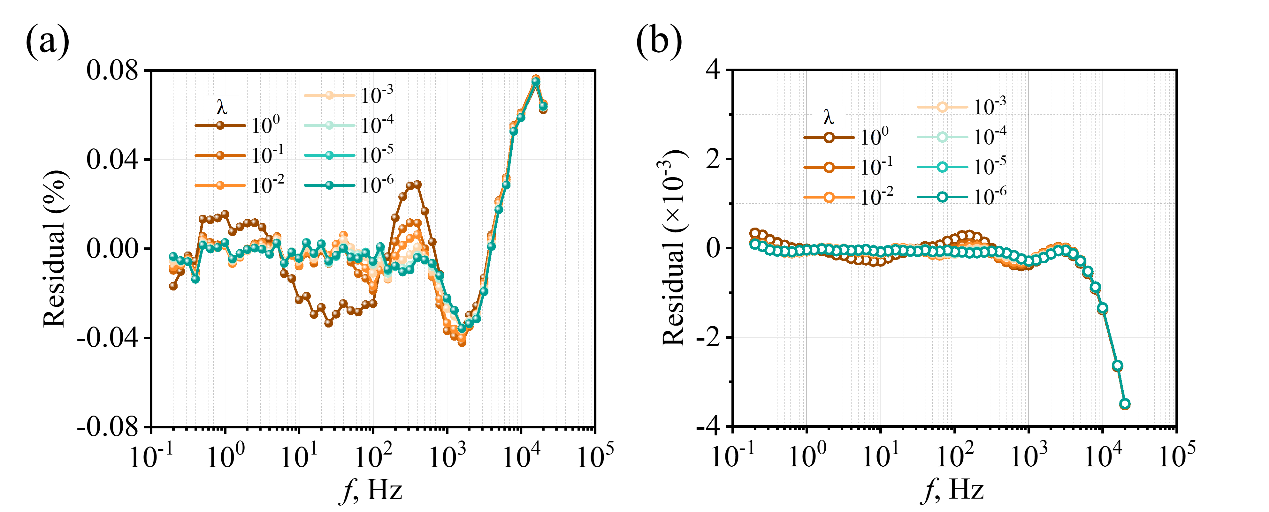


Fig. S6 residual distribution as a function of frequency for different values of *λ*: (a) Real part;

(b) Imaginary part.

# S4 Assignment of the DRT function peaks

Table S1 The definition of different cases under various MEA component and operating conditions

|  | Mass flow rate (L/min) | | Catalyst loading (mg/cm^2^) | | Membrane  (um) |
| --- | --- | --- | --- | --- | --- |
|  | Anode | Cathode | Anode | Cathode |  |
| Reference case | 0.4 | 0.5 | 0.6 | 0.4 | 20 |
| Case 1 | **0.04** | 0.5 | 0.6 | 0.4 | 20 |
| Case 2 | 0.4 | **0.05** | 0.6 | 0.4 | 20 |
| Case 3 | 0.4 | 0.5 | **0.3** | 0.4 | 20 |
| Case 4 | 0.4 | 0.5 | 0.6 | **0.2** | 20 |
| Case 5 | 0.4 | 0.5 | 0.6 | 0.4 | 20 |
| Case 6 | 0.4 | 0.5 | 0.6 | 0.4 | **40** |


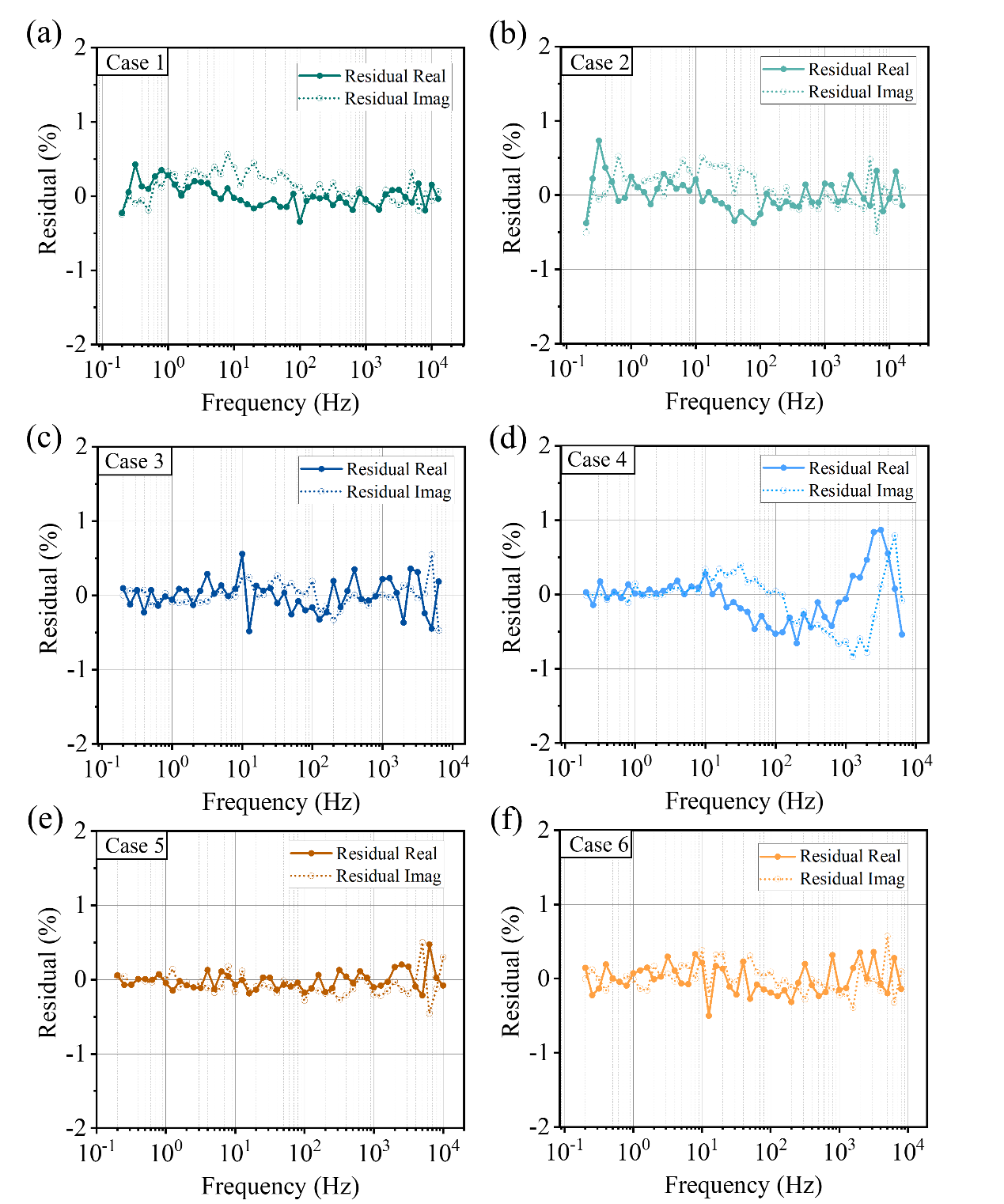


Fig. S7 Relative residual of the KK transformation of different cases. The defination of Case1-Case 6 is provided in table S1


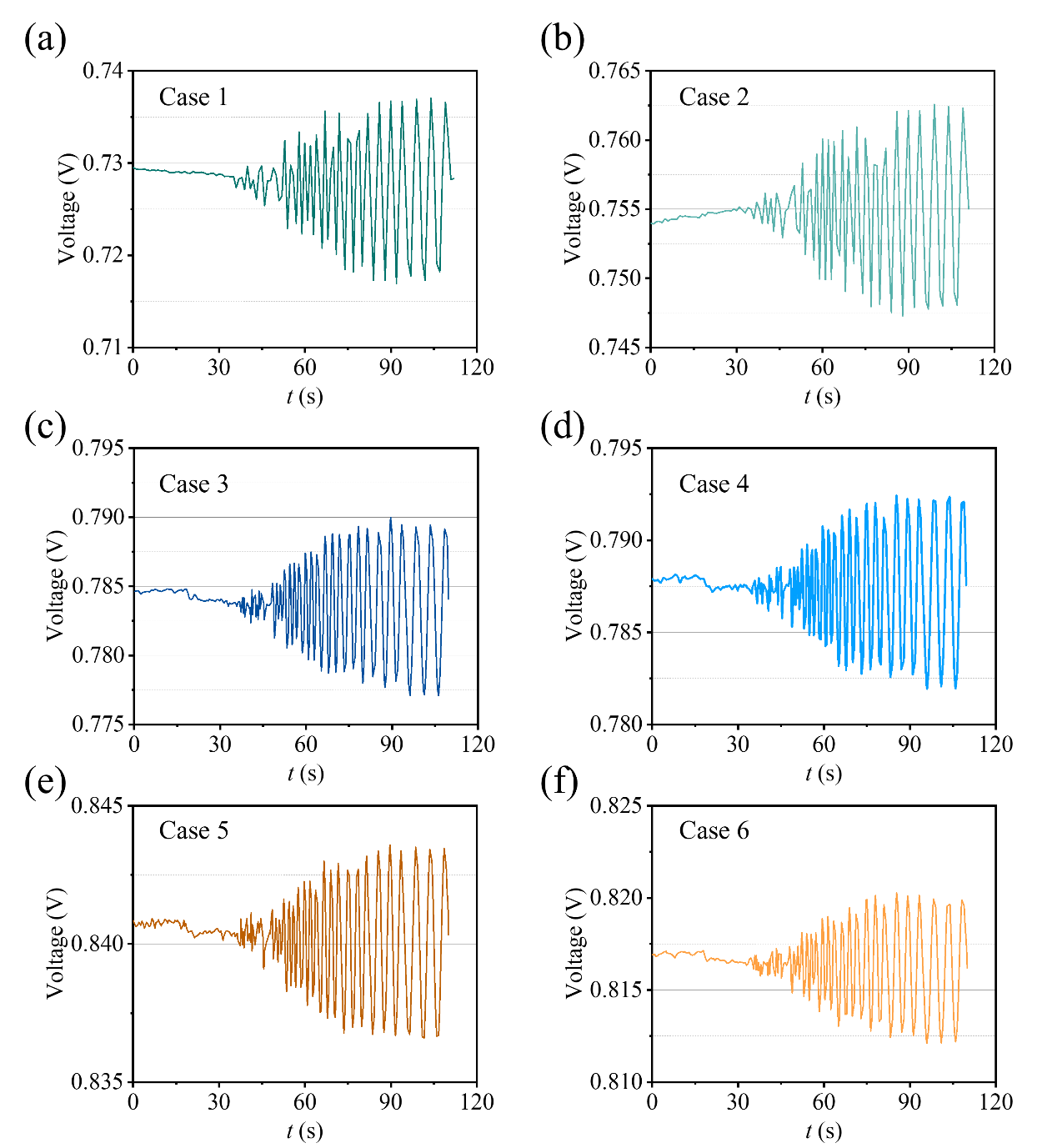


Fig. S8 The relationship *i* and *V*_cell_ as a function of *t* during the EIS testing, where cell voltage variation is solely caused by the current perturbation. The defination of Case1-Case 6 is provided in table S1
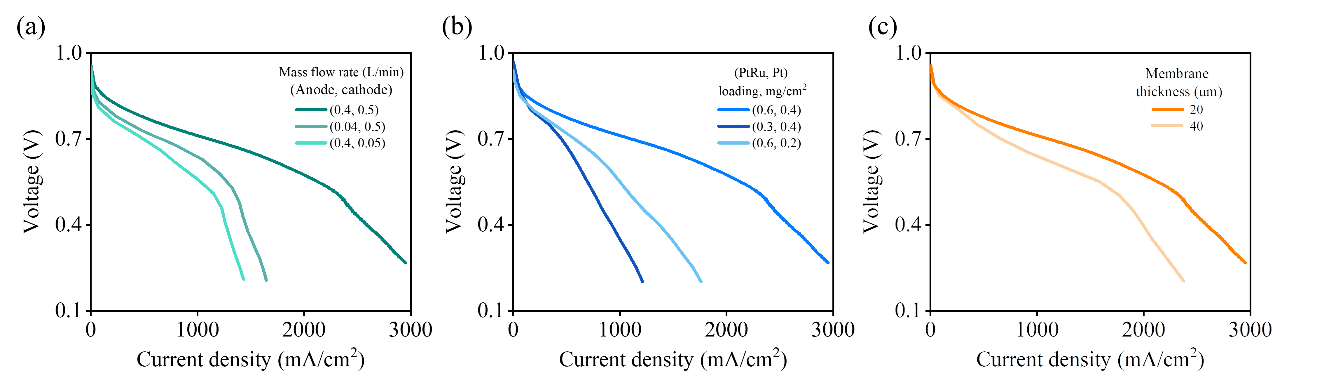


Fig. S9 Polarization curves under different MEA component and operating conditions


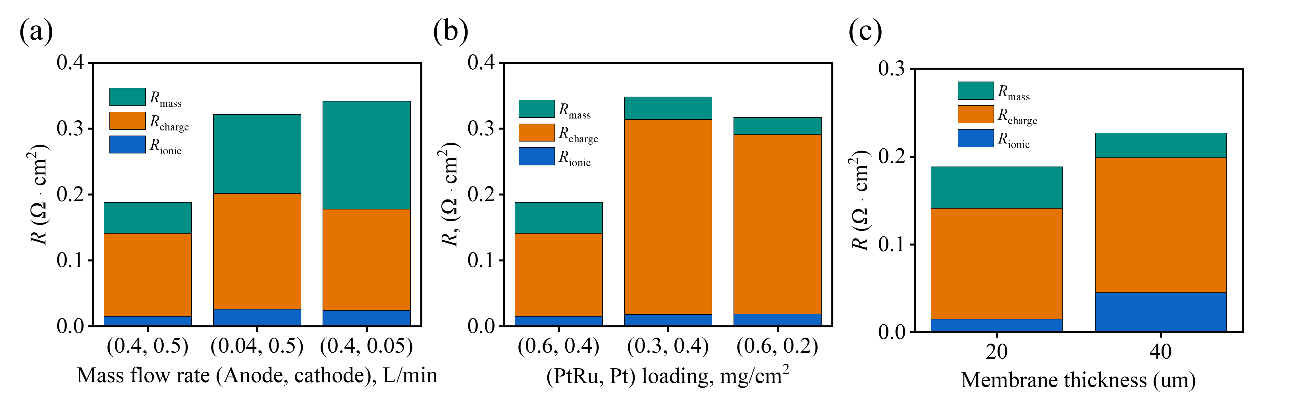


Fig. S10 Bar graphs show the integration results of DRT curves across different frequency ranges under different MEA component and operating conditions

# S5 Effect of current density


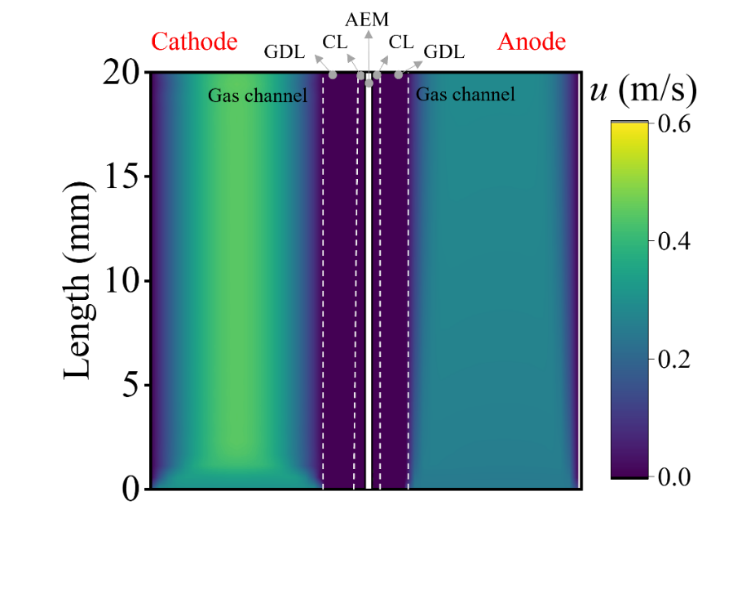


Fig. S11 Fluid flow velocity distribution of AEMFCs at *i* = 1800 mA/cm^2^ under baseline operating conditions

# S6 Effect of relative humidity


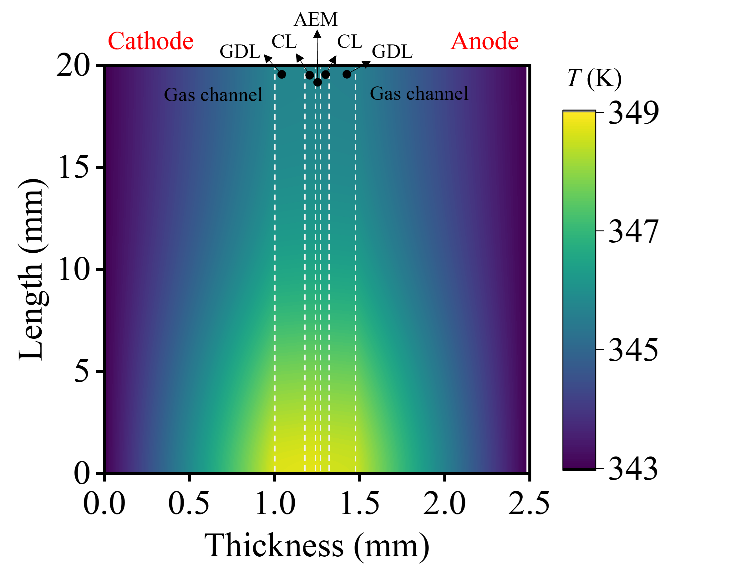


Fig. S12 Temperature distribution of AEMFCs at *i* = 1800 mA/cm^2^ under baseline operating conditions

# S7 Durability testing


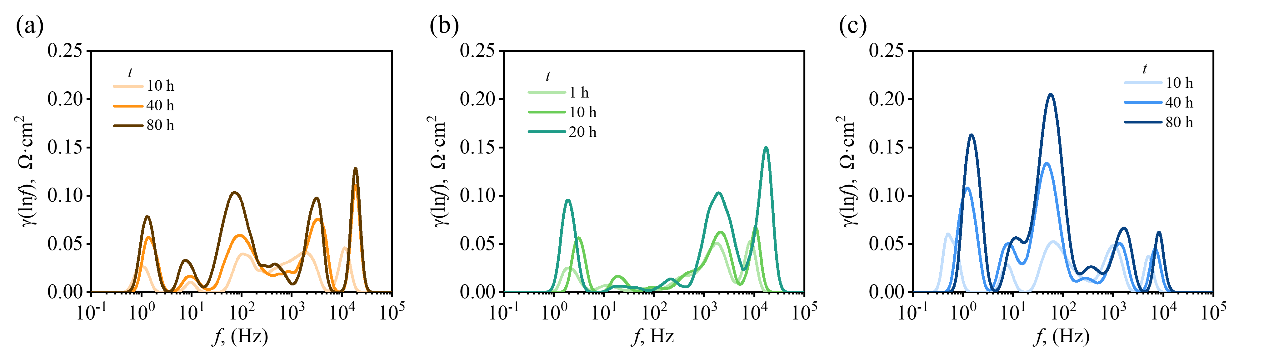


Fig. S13 DRT plots at different testing time point under three water content conditions:

(a) Normal; (b) Cathode drying; (c) Anode flooding
